# AJUBA promotes the migration and invasion of esophageal squamous cell carcinoma cells through upregulation of MMP10 and MMP13 expression

**DOI:** 10.18632/oncotarget.9239

**Published:** 2016-05-09

**Authors:** Xuejiao Shi, Zhaoli Chen, Xueda Hu, Mei Luo, Zengmiao Sun, Jiagen Li, Susheng Shi, Xiaoli Feng, Chengcheng Zhou, Zitong Li, Wenhui Yang, Yuan Li, Pan Wang, Fang Zhou, Yibo Gao, Jie He

**Affiliations:** ^1^ Department of Thoracic Surgery, Cancer Institute and Hospital, Peking Union Medical College and Chinese Academy of Medical Sciences, Beijing, China; ^2^ Department of Pathology, Cancer Institute and Hospital, Peking Union Medical College and Chinese Academy of Medical Sciences, Beijing, China

**Keywords:** AJUBA, ERK1/2, MMP10, MMP13, ESCC

## Abstract

The LIM-domain protein AJUBA has been reported to be involved in cell-cell adhesion, proliferation, migration and cell fate decision by acting as a scaffold or adaptor protein. We previously identified AJUBA as a putative cancer gene in esophageal squamous cell carcinoma (ESCC). However, the function and underlying mechanisms of AJUBA in ESCC remain largely unknown. In the present study, we detected AJUBA levels in ESCC tumor tissues and in corresponding adjacent non-tumor tissues by immunohistochemistry (IHC) and investigated the function and mechanism of AJUBA in ESCC cells. The IHC results showed that AJUBA levels were significantly higher in ESCC tissues compared with corresponding adjacent non-tumor tissues (*P* < 0.001). Both *in vitro* and *in vivo* experiments showed that AJUBA promoted cell growth and colony formation, inhibited cisplatin-induced apoptosis of ESCC cells, and promoted ESCC cell migration and invasion. RNA sequencing was used to reveal the oncogenic pathways of AJUBA that were involved, and MMP10 and MMP13 were identified as two of the downstream targets of AJUBA. Thus, AJUBA upregulates the levels of MMP10 and MMP13 by activating ERK1/2. Taken together, these findings revealed that AJUBA serves as oncogenic gene in ESCC and may serve as a new target for ESCC therapy.

## INTRODUCTION

Esophageal cancer is the sixth leading cause of cancer death and the eighth most frequently diagnosed cancer worldwide [1, 2], and most esophageal cancer cases are esophageal squamous cell carcinoma [3]. Few significant improvements in the overall survival of ESCC patients have been achieved; the 5-year overall survival rate remains poor due to the advanced stage at initial diagnosis and due to a lack of effective therapies [4]. The identification of molecular mechanisms critical for driving ESCC progression is much needed.

AJUBA family proteins (AJUBA, LIMD1 and WTIP) belong to the Zyxin/AJUBA family. These proteins are characterized by the conservation of three tandem C-terminal LIM domains and a unique N-terminal preLIM region, which includes a nuclear export signal (NES) [5]. AJUBA family proteins can function as negative regulators of the Hippo pathway, affecting cell proliferation and controlling tissue size [6, 7]. Moreover, AJUBA can regulate many other cellular events, such as the meiotic maturation of oocytes [5], response to DNA damage [8], modulation of the actin cytoskeleton [9], and migration and invasion of cells [10]. Increasing evidence, including the detection of AJUBA mutations in multiple human cancers such as ESCC [11, 12], cutaneous squamous cell carcinoma [13] and head and neck squamous cell carcinomas [14], suggests a role for AJUBA in tumorigenesis. However, previous studies have primarily focused on the *Drosophila* homolog of AJUBA [6, 7, 15], and the role of AJUBA in human cancer development has been controversially reported [10, 16].

In the present study, we detected the expression levels of AJUBA by IHC and performed both *in vitro* and *in vivo* functional assays to characterize the biological effects of AJUBA on ESCC tumorigenicity and metastasis. The oncogenic mechanism of AJUBA was also investigated.

## RESULTS

### AJUBA was frequently overexpressed in ESCC

**Figure 1:**
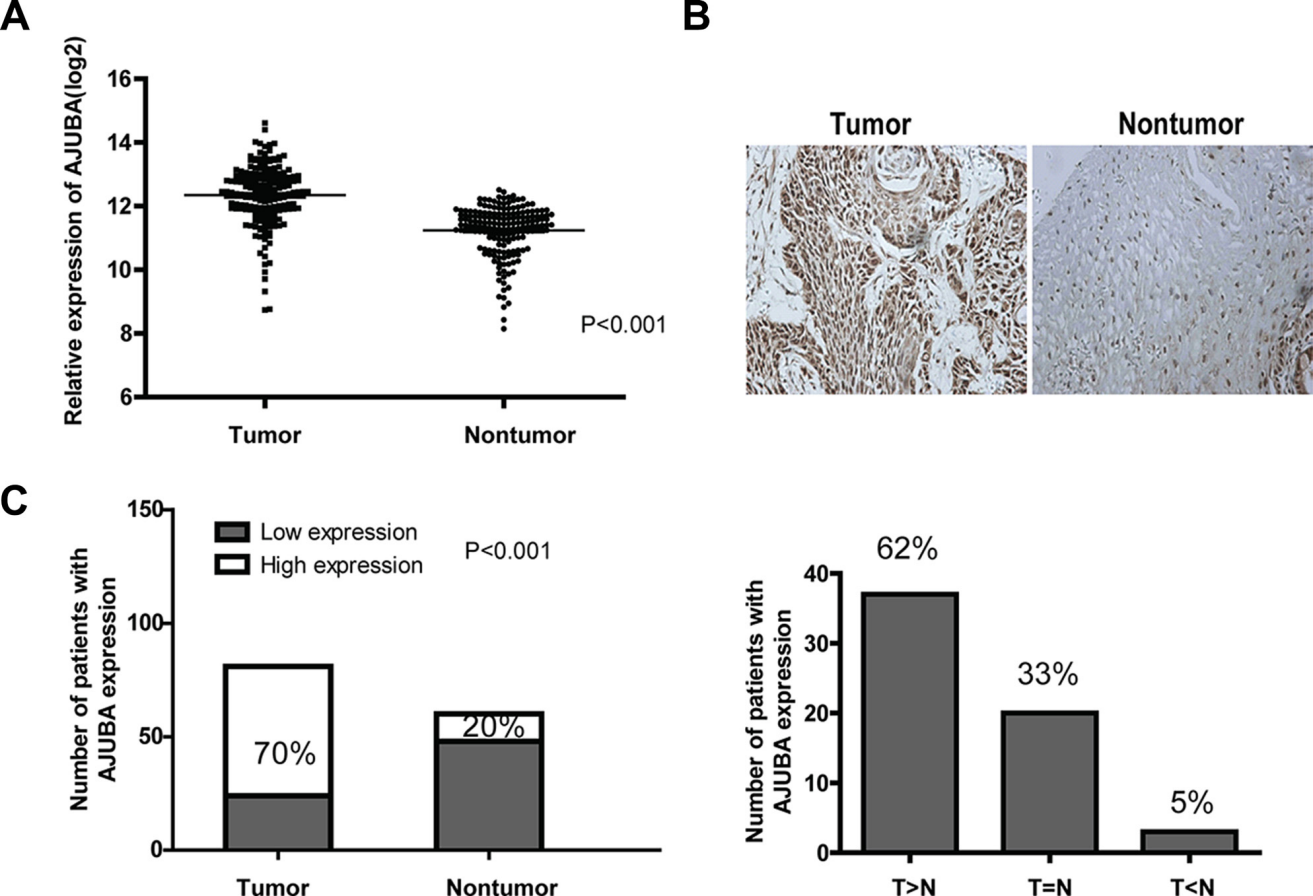
AJUBA was frequently upregulated in ESCC tissues compared with non-tumor tissues (**A**) Analysis of AJUBA mRNA level according to our previous microarray data (*n* = 179). *P* < 0.001, paired Student's *t*-test. (**B**) Detection of the protein level of AJUBA in a paraffin-embedded formalin-fixed ESCC tissue microarray containing 81 tumors and 60 corresponding non-tumor tissues by IHC. Representative images of AJUBA in tumor tissues and non-tumor tissues. (**C**) The number of patients with ESCC expressing low (negative and weak positive staining) and high (moderate and strong positive staining) levels of AJUBA in tumor tissues and non-tumor tissues (left panel). *P* < 0.001, χ^2^ test. AJUBA expression in 60 paired ESCC tissues (right panel). T: tumor tissue; N: non-tumor tissue.

Previously, through exome sequencing, we identified AJUBA somatic mutations in ESCC [11]. Here, we analyzed the mRNA levels of AJUBA and two other AJUBA family members, WTIP and LIMD1, in ESCC tumor tissues and in their matched adjacent non-tumor tissues. From 179 paired samples, we found that AJUBA was significantly overexpressed in tumor tissues than in adjacent non-tumor tissues (mean, 2.15-fold; *P* < 0.001, paired Student's *t*-test, two-tailed) (Figure 1A). However, the mRNA levels of WTIP and LIMD1 were not dysregulated in tumor tissues (Supplementary Figure S1). The AJUBA protein levels were then detected by IHC in a tissue microarray (TMA) containing 81 primary esophageal tumor tissues and 60 corresponding non-tumor tissues from the same cohort as that of the RNA microarray (Figure 1B). Overall, 70% (57/81) of tumor tissues showed high expression of AJUBA (moderate to strong staining), whereas only 20% (12/60) of non-tumor tissues had high expression of AJUBA; this difference was statistically significant (*P* < 0.001, χ^2^ test). When comparing the staining result of tumor tissues with their paired non-tumor tissues, 62% (37/60) of the tumor tissues exhibited increased AJUBA expression (Figure 1C). These results indicated that AJUBA was frequently overexpressed in ESCC tumor tissues. Moreover, the results showed that in non-tumor tissues, 38% AJUBA positive cases showed nucleus staining, 62% AJUBA positive cases showed cytoplasm staining. While in tumor tissues, only 2% AJUBA positive cases had nucleus staining, 86% AJUBA positive cases had cytoplasm staining, and the remaining 12% cases had both nucleus and cytoplasm staining.

Next, the relationships between AJUBA expression in ESCC tissues and clinicopathological characteristics were analyzed in 81 patients with ESCC. In this cohort, expression level of AJUBA was associated with tumor cell differentiation (*P* = 0.043, χ^2^ test) and invasion depth (T stage, *P* = 0.005, Fisher's exact test). Furthermore, patients with high AJUBA expression had poorer differentiation and a higher tumor grade (Table 1).

**Table 1:** The relationships between AJUBA levels and clinicopathological characteristics in ESCC tissues

Characteristics	Total (*n* = 81)	AJUBA expression		*P* value
		Low	High	
Age				
< 60	39	14	25	0.234
≥ 60	42	10	32	
Gender				
Female	14	4	10	1.000
Male	67	20	47	
Smoking				
No	36	12	24	0.514
Yes	45	12	33	
Drinking				
No	35	10	25	0.856
Yes	46	14	32	
Family history				
No	71	21	50	1.000
Yes	10	3	7	
Differentiation				
High	16	7	9	0.043
Middle	44	15	29	
Low	21	2	19	
T Classification				
T1	6	3	3	0.005
T2	12	4	8	
T3	53	10	43	
T4	10	7	3	
N classification				
N < 1	36	11	25	0.870
N ≥ 1	45	13	32	
TNM stage				
I	4	3	1	0.076
II	35	7	28	
III	41	14	27	
IV	1	0	1	

### AJUBA knockdown inhibited tumor growth *in vitro* and *in vivo*

**Figure 2:**
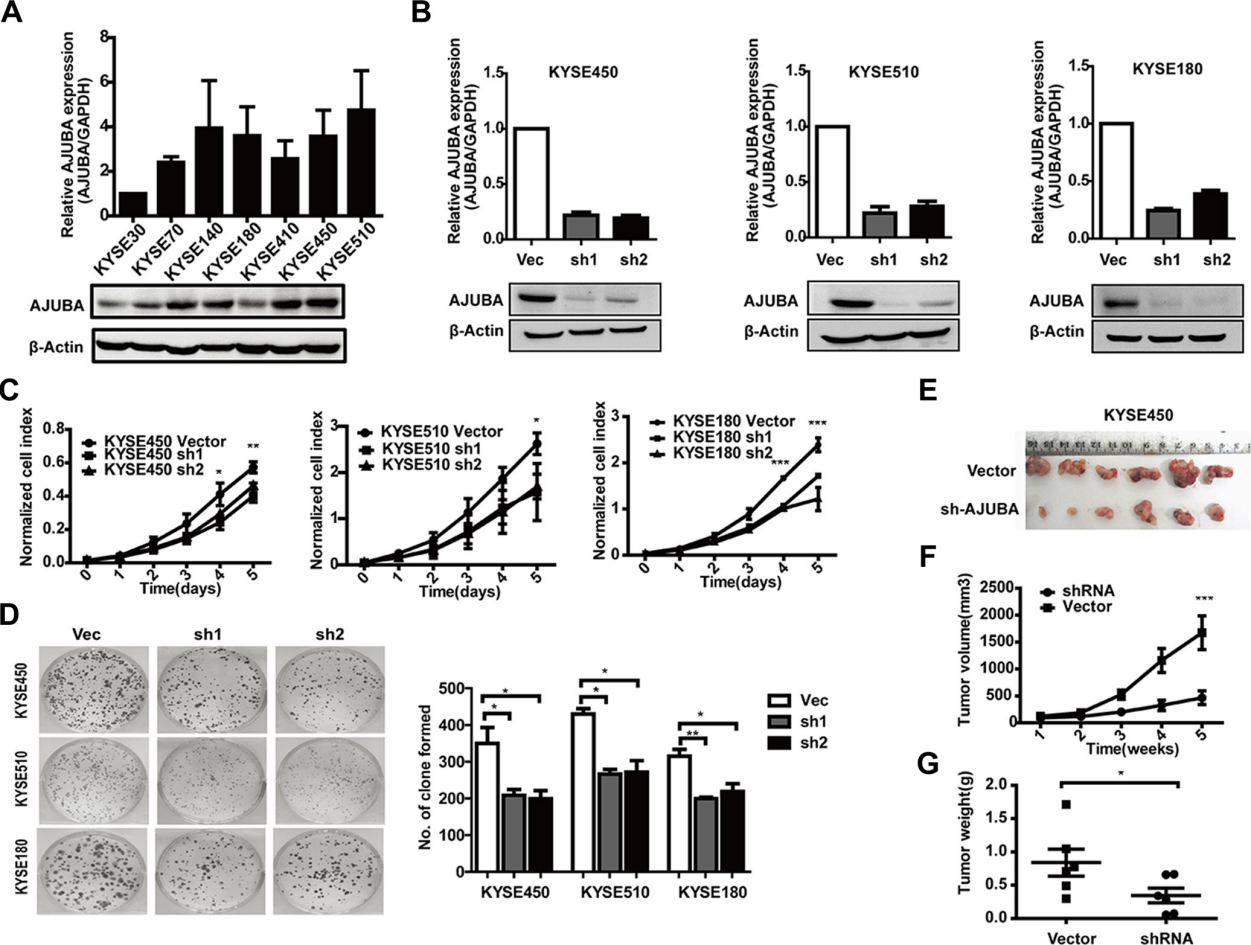
AJUBA knockdown in ESCC cells suppressed cell proliferation *in vitro* and *in vivo* (**A**) The mRNA and protein levels of AJUBA were detected in 7 ESCC cell lines by RT-PCR and Western blot. (**B**) RT-PCR and Western blot analyses were used to detect the knockdown efficiency of AJUBA. GAPDH and β-Actin were used as controls. (**C**) The proliferation of KYSE450, KYSE510 and KYSE180 cells treated with shRNA or empty vector was measured by CCK8 assay at different time intervals ranging from 0 to 120 h. The results are shown as the mean ± SEM of three independent experiments. *P* values were obtained using two-way ANOVA. (**D**) Representative inhibition of clone formation in 6-well plates by shAJUBA compared with control cells. The columns show the mean number of clones formed in three independent experiments. *P < 0.05; **P < 0.01 based on Student's *t*-test. (**E**, **F**, and **G**) AJUBA knockdown markedly reduced the volume and weight of the tumor mass. *P* values were obtained using paired *t*-tests.

First, we measured AJUBA mRNA and protein levels in 7 ESCC cell lines by RT-PCR and Western blot (Figure 2A). KYSE450, KYSE510 and KYSE180 ESCC cell lines with relatively higher levels of AJUBA expression were used in further RNA interference studies. We then generated two shRNAs that could dramatically decrease both the mRNA and protein levels of AJUBA (Figure 2B) and evaluated the effect of AJUBA on cell proliferation. The results showed that AJUBA depletion significantly inhibited the proliferation (Figure 2C) and colony formation of KYSE450, KYSE510 and KYSE180 cells (Figure 2D). To further investigate the effect of AJUBA on tumor growth, 5 × 10^6^of AJUBA-depleted or control KYSE450 cells were *subcutaneously* inoculated into the left or right dorsal flanks of female BALB/c-nu mice, respectively. The size (Figure 2E and 2F) and weight (Figure 2G) of tumors were significantly reduced in AJUBA knockdown mice compared with the control group (*P* < 0.05, paired *t*-tests). Similar results were also observed for KYSE510 cells (Supplementary Figure S2). These results indicated that AJUBA promotes ESCC cell proliferation and tumor formation *in vitro* and *in vivo*.

**Figure 3:**
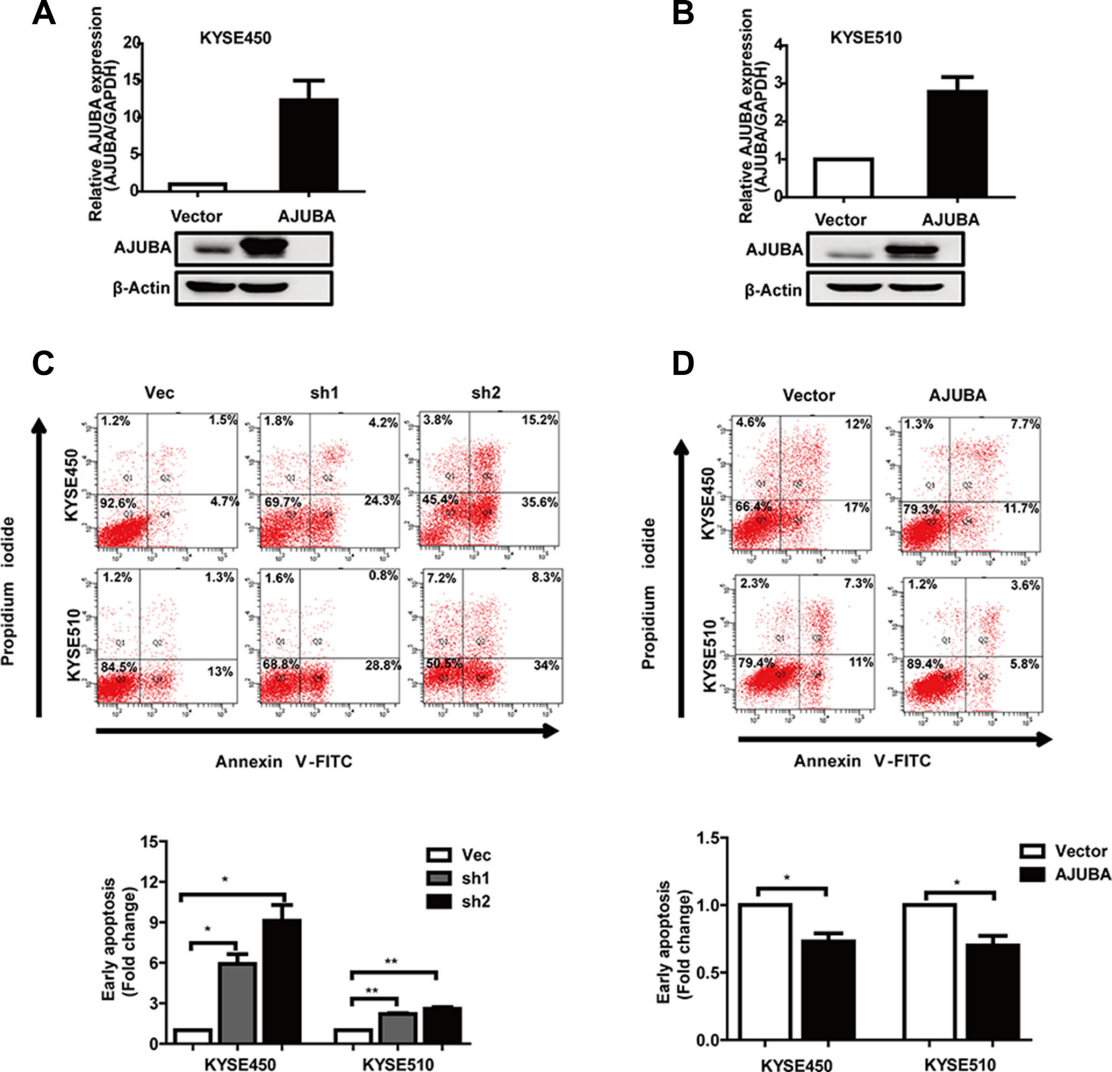
AJUBA inhibited the cisplatin-induced apoptosis of ESCC (**A** and **B**) KYSE450 and KYSE510 cells were transfected with the AJUBA CDS or empty vector, and the mRNA and protein levels of AJUBA were confirmed by RT-PCR and Western blot. (**C**) KYSE450 and KYSE510 cells transfected with shRNA or control cells. (**D**) Vec-KYSE450, Vec-KYSE510 or AJUBA-KYSE450, AJUBA-KYSE510 cells were seeded, cultured in 6-well plates for 24 h and harvested 24 h after treatment with cisplatin. Early apoptosis was measured with FACS-based annexin-V/PI double staining. The results are expressed as the mean ± SEM. *P < 0.05; **P < 0.01, Student's *t*-test.

To determine whether AJUBA affects ESCC tumor cell survival, we treated KYSE450 and KYSE510 cells with cisplatin for 24 hours. The flow cytometric analyses showed that after 24 hours of cisplatin treatment, AJUBA knockdown significantly enhanced the apoptosis of KYSE450 and KYSE510 cells (Figure 3C). In contrast, AJUBA overexpression rendered ESCC cells more resistance to cisplatin-induced apoptosis (Figure 3D). These data suggested that regulation of AJUBA expression might sensitize the ESCC cell response to chemotherapeutic drugs, such as cisplatin.

### AJUBA overexpression enhanced tumor migration and invasion *in vitro* and *in vivo*

**Figure 4:**
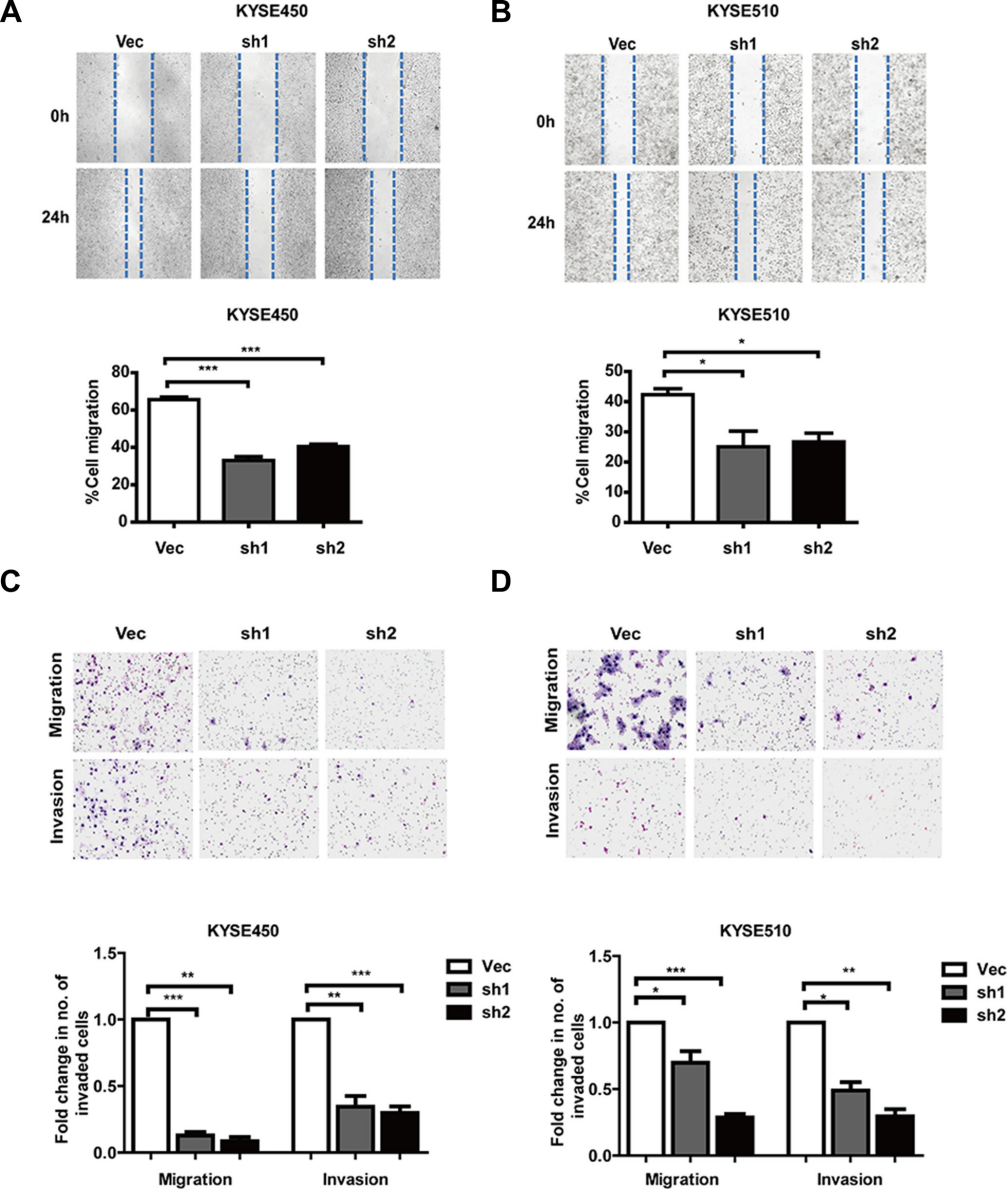
Downregulation of AJUBA inhibited cell motility and invasiveness (**A** and **B**) Wound-healing assays were performed to compare the migration of shRNA-treated cells and control cells. The results are shown as the mean ± SEM of three independent experiments. *P < 0.05; **P < 0.01; ***P < 0.001, Student's *t*-test. (**C** and **D**) Transwell assays were used to compare migration and invasion between shAJUBA and control cells. The cells that migrated through the membrane or invaded the lower surface of the membrane were fixed and stained with Giemsa. Student's *t*-test was used to analyze the results.

**Figure 5:**
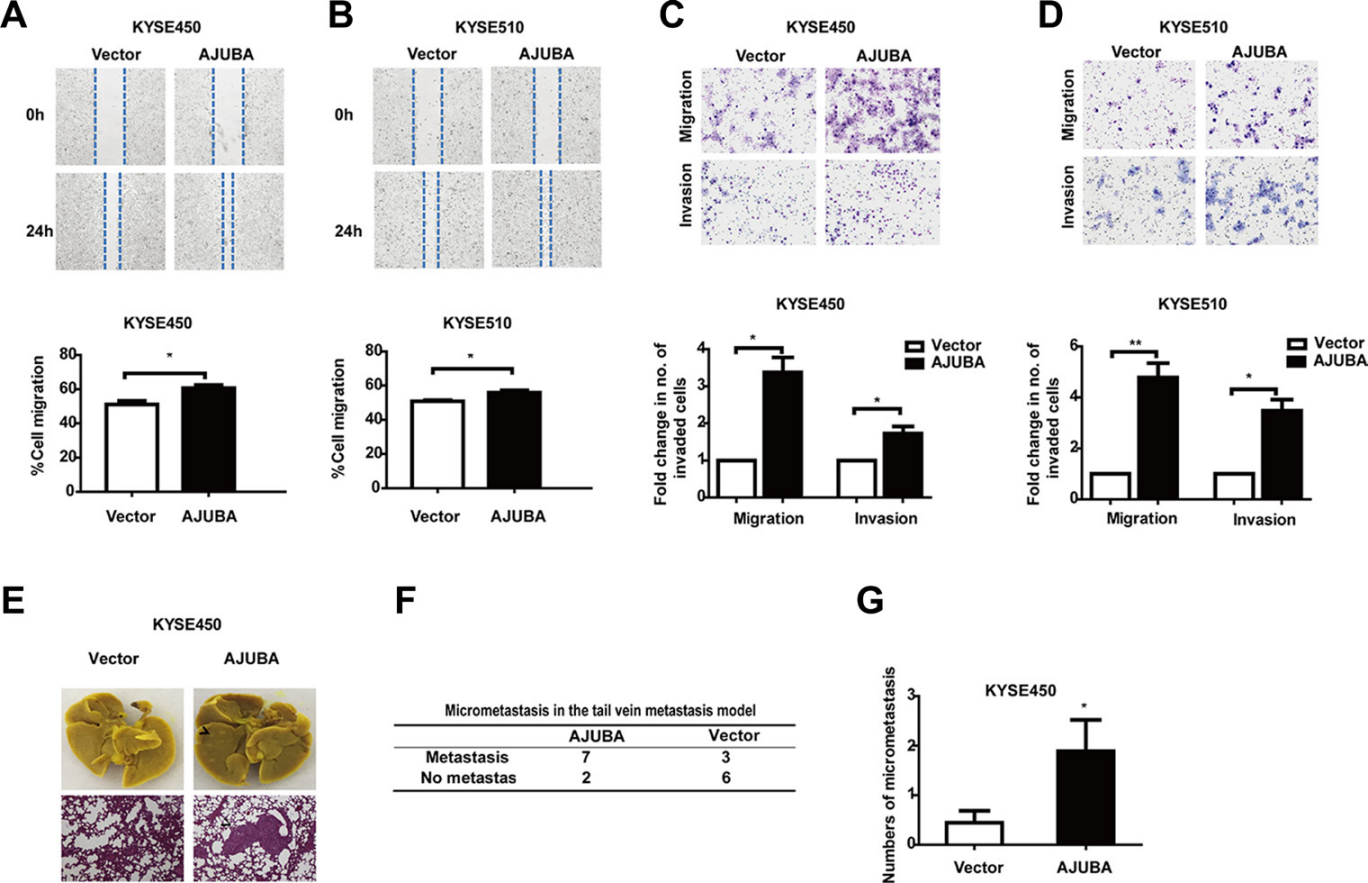
AJUBA overexpression promoted cell motility and invasiveness *in vitro* and *in vivo* (**A** and **B**) Wound-healing assays were performed to compare the migration of AJUBA CDS-transfected cells and control cells. The results are shown as the mean ± SEM of three independent experiments. *P < 0.05; **P < 0.01, Student's *t*-test. (**C** and **D**) Transwell assays were used to compare migration and invasion between AJUBA CDS-transfected cells and control cells. Student's *t*-test was used to analyze the results. (**E**) Representative images of isolated lungs (upper) and H&E-stained lung sections (lower) from each group are shown. (**F**) The number of mice with micrometastases is shown. (**G**) The number of lung micrometastases in NOD-SCID mice with i.v. injection of KYSE450-AJUBA or control cells was counted under a microscope. *P < 0.05, Student's *t*-test.

We next evaluated the effect of AJUBA on ESCC cell motility. The results of wound-healing and Transwell assays showed that AJUBA depletion significantly inhibited the migration and invasion of KYSE450 and KYSE510 cells (Figures 4A–4D). In contrast, exogenous expression of AJUBA significantly increased the migratory and invasive abilities of KYSE450 and KYSE510 cells (Figures 5A–5D). Moreover, AJUBA overexpression can also increase the motility of KYSE30 and KYSE70 cell lines which with low endogenous AJUBA levels (Supplementary Figure S3).

To further evaluate the effect of AJUBA expression on ESCC cell metastasis, 1 × 10^6^ KYSE450 cells that stably overexpressed AJUBA were intravenously injected into non-obese diabetic (NOD)-SCID mice. The number of micrometastatic nodules in the lungs was counted 12 weeks later by histomorphological analysis under a microscope (Figure 5E). Micrometastatic nodules in the lungs were observed in 7 of 9 mice injected with cells overexpressing AJUBA, whereas only 3 of 9 mice in the control group had lung micrometastatic nodules (Figure 5F). Moreover, the numbers of micrometastatic nodules in the AJUBA overexpression group were significantly higher than those in the control group (Figure 5G, *P* < 0.05, Student's *t*-test).

### AJUBA upregulated MMP10 and MMP13 expression in ESCC cells

**Figure 6:**
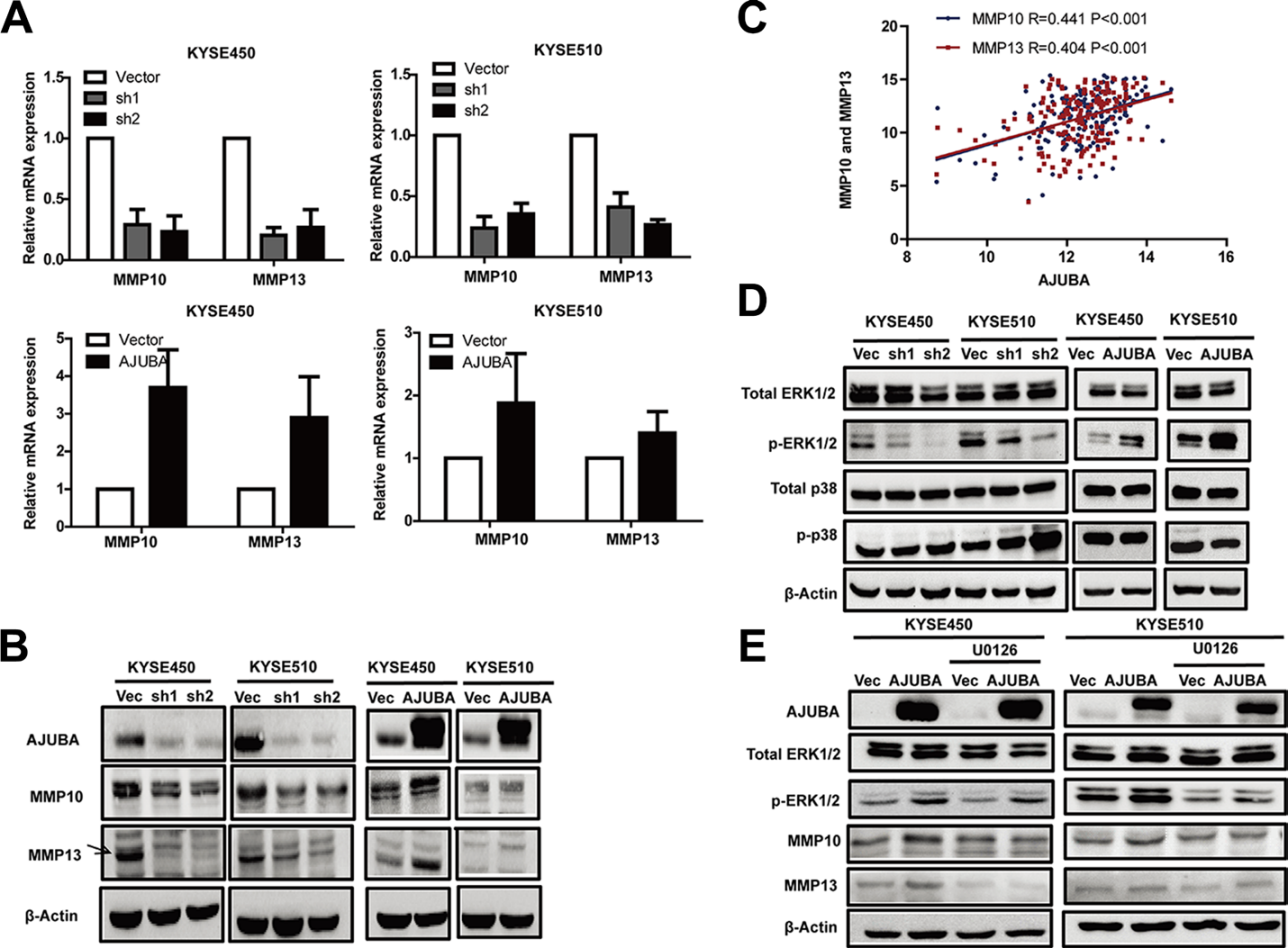
AJUBA upregulated the expression of MMP10 and MMP13 via the ERK1/2 signaling pathway (**A**) The relative mRNA levels of MMP10 and MMP13 in AJUBA-knockdown cells or AJUBA-overexpression cells compared with control cells were detected by RT-PCR. GAPDH was used as a control. (**B**) Western blot was used to detect the expression of MMP10 and MMP13. β-Actin was used as a loading control. (**C**) Correlation between the mRNA level of AJUBA and MMP10 and MMP13 mRNA expression in ESCC cells. (**D**) Total ERK1/2, p-ERK1/2, total p38 and p-p38 levels were detected by Western blot. β-Actin was used as a loading control. (**E**) The expression levels of AJUBA, total ERK1/2, p-ERK1/2, MMP10 and MMP13 were compared between cells treated with U0126 (20 μmol) for 24 h and control cells by Western blot. β-Actin was used as a loading control.

We next explored the molecular events that were involved in the AJUBA-mediated downstream regulatory network. RNA sequencing was used to identify the altered gene expression profiles following AJUBA knockdown in KYSE450, KYSE510 and KYSE180 cells. Genes that were significantly upregulated or downregulated (2-fold, *P* < 0.01, FDR < 0.1) by AJUBA knockdown in three cell lines were selected for Gene Ontology (GO) analysis. The GO analysis revealed that a number of genes involved in cell motility, cell adhesion and cell junctions were significantly dysregulated following AJUBA knockdown (Supplementary Figure S4). Among these genes, the mRNA levels of MMP10 and MMP13 were downregulated by 5.6-fold and 5.5-fold, respectively, in AJUBA-depleted cells compared with the control cells (Supplementary Table S1). The positive correlation between AJUBA and MMP10 and MMP13 expression was confirmed by RT-PCR (Figure 6A) and Western blot (Figure 6B) after AJUBA knockdown or overexpression in KYSE450 and KYSE510 cells. In addition, AJUBA mRNA levels were significantly associated with elevated MMP10 and MMP13 expression in 179 ESCC tumor tissues (*r* = 0.441, *P* < 0.001 and *r* = 0.404, *P* < 0.001, respectively, Figure 6C and Supplementary Table S2), suggesting that AJUBA promotes the expression of MMP10 and MMP13 in ESCC.

### AJUBA enhanced MMP10 and MMP13 expression by activating ERK1/2

The mitogen-activated protein kinase (MAPK) signaling pathway, including the well-known mediator extracellular signal-regulated kinase 1/2 (ERK1/2), regulates MMP expression in different cancer types [17–20]. A previous study reported that AJUBA could augment MAPK activity by interacting with Grb2 [5]. To investigate the molecular mechanism by which AJUBA promoted MMP10 and MMP13 expression in ESCC cells, we examined the effects of AJUBA on ERK1/2 activation. The Western blot analysis showed that the level of phosphorylated ERK1/2, but not p38 MAPK, was dramatically decreased in ESCC cells with AJUBA knockdown and increased in ESCC cells with AJUBA overexpression (Figure 6D and Supplementary Figure S3). To further investigate the key role of ERK1/2 in AJUBA-mediated regulation of MMP10 and MMP13 expression, U0126, the small molecule inhibitor of MEK1/2, was used to suppress the phosphorylation of ERK1/2 in ESCC cells. Treatment with U0126, which largely decreased the level of phosphorylated ERK1/2, reduced MMP10 and MMP13 expression in KYSE450 and KYSE510 cells (Figure 6E). In addition, U0126 attenuated the effect of AJUBA overexpression on the upregulation of MMP10 and MMP13. These data suggested that AJUBA enhances MMP10 and MMP13 expression in an ERK1/2-dependent manner.

## DISCUSSION

AJUBA, which was originally identified using a yeast two-hybrid screen, participates in different signaling pathways by interacting with a number of proteins via the LIM domain or preLIM region [21–25]. Previous studies have indicated that AJUBA promotes cell proliferation by inhibiting the Hippo/Yes-associated protein (YAP) pathway [6, 26], and Liang et al. reported that the AJUBA level is upregulated in colorectal cancer tissues [10]. Consistent with these findings, the data obtained in the present study showed that the level of AJUBA, but not WTIP nor LIMD1, was significantly increased in ESCC tissues compared with non-tumor tissues. In line with these clinical data, AJUBA knockdown effectively inhibited cell growth, colony formation, and tumor formation. Platinum-based chemotherapy is the first-line agent for patients with advanced ESCC, and apoptosis-related genes, such as TP53, are ubiquitously mutated in ESCC [11]. In this study, down-regulation of AJUBA alleviated the resistance of ESCC cells to cisplatin, suggesting that AJUBA can be target for the clinical treatment of ESCC.

MMP10 and MMP13 can degrade the extracellular matrix and promote cancer metastasis and progression [27–33]. Overexpression of MMP10 and MMP13 in ESCC tissues has been shown in several studies [34, 35]. In this study, MMP10 and MMP13 were identified as the downstream targets of AJUBA by RNA-sequencing. AJUBA promoted ESCC migration and invasion through upregulating MMP10 and MMP13. The correlation analysis between AJUBA and MMP10 and MMP13 in 179 ESCC tissues further confirmed the results and strengthened the importance of AJUBA in ESCC progression. MAPK/ERK1/2 pathways play critical roles in cell growth, apoptosis and metastasis [36], and several studies reported that MMP activities could be affected by the level of p-ERK1/2 in ESCC [19, 37]. Consistent with these findings, we found that AJUBA promoted the expression of MMP10 and MMP13 partially through upregulating the MAPK/ERK1/2 pathway.

Recently, AJUBA mutations were found in many types of tumors [11–14]. Most of these mutations were insertions, deletions or splice-site alterations, and truncating mutations of AJUBA can promote ESCC progression [12]. Truncations and subcellular localization can have distinct effects on the function of AJUBA and on the cellular activities of tumor cells. The overexpression of full-length AJUBA or of the AJUBA preLIM domain increased the proliferation of P19 embryonal carcinoma cells, whereas overexpression of the three LIM domains of AJUBA reduced cell growth. In addition, AJUBA localized to the nucleus of normal mouse lung epithelial cells and mainly localized to the cytoplasm of tumor tissues of malignant mesothelioma [16, 38]. Furthermore, the accumulation of the LIM domains of AJUBA in the nucleus resulted in growth inhibition and spontaneous endodermal differentiation [39]. Consistent with these findings, our study showed that AJUBA mainly localized in the cytoplasm in tumor tissues, and the percentage of nucleus staining dramatically decreased in tumor tissues compared with non-tumor tissues, suggesting that AJUBA may transfer from nucleus to cytoplasm and different domains of AJUBA and the subcellular location of AJUBA affect the subcellular activities of AJUBA and, thus, tumor cell functions.

In conclusion, our study revealed that AJUBA was frequently upregulated in ESCC tumor tissues. AJUBA overexpression promoted the tumorigenicity and motility of ESCC and upregulated MMP10 and MMP13 expression by increasing the level of pERK1/2. The deletion of AJUBA decreased the resistance of ESCC cells to cisplatin, which could serve as a new strategy for ESCC treatment.

## MATERIALS AND METHODS

### Cell lines and clinical samples

The esophageal cancer cell lines KYSE30, KYSE70, KYSE140, KYSE180, KYSE410, KYSE450 and KYSE510 were grown in RMPI 1640 medium (HyClone, Logan, UT, USA). HEK 293T cells were maintained in DMEM medium (HyClone). All culture media were supplemented with 10% fetal bovine serum (Gibco, New York, NY, USA), 100 UI/ml penicillin and 100 UI/ml streptomycin. All cell lines used in this study were regularly authenticated by short tandem repeat (STR) detection and tested for the absence of mycoplasma contamination. The clinical ESCC samples used in this study were histopathologically and clinically diagnosed at the Cancer Institute and Hospital of the Chinese Academy of Medical Science with written consent and approval from the institutional research ethics committee.

### Real-time polymerase chain reaction (RT-PCR)

Total RNA was isolated from cultured cell lines using TRIzol reagent (Invitrogen, Carlsbad, CA, USA), and complementary DNA (cDNA) was synthesized using a Fermentas RevertAid^™^ Premium First Strand cDNA Synthesis Kit (Thermo, Rockford, IL, USA). Quantitative RT-PCR (qPCR) was performed in triplicate using SYBR Green PCR master mix and an Applied Biosystems 7300 or 7900 RT-PCR System (Applied Biosystems, Foster City, CA, USA) following the manufacturer's instructions. The 2^−ΔΔCt^ method was used to quantify the expression levels relative to GAPDH expression. Three independent experiments were carried out to analyze the relative mRNA expression level, and each sample was tested in triplicate. The primers used in the study are provided in Supplementary Table S3.

### Western blot

The cultured cells were lysed in RIPA buffer supplemented with protease and phosphatase inhibitor mixtures (Thermo). Equal amounts of proteins were separated by 10% SDS–PAGE and then transferred to nitrocellulose filter membranes (Whatman GmbH, Maidstone, Kent, UK). The membranes were blocked at room temperature for 1 h with 5% non-fat milk and subsequently incubated with primary antibodies directed against AJUBA, ERK1/2, p-ERK1/2 (Cell Signaling Technology, Danvers, MA, USA), MMP10 and MMP13 (Abcam, Cambridge, UK) at 4°C overnight. β-Actin (Epitomics, Hangzhou, China) was used as a loading control. After washing with TBST, the blots were incubated with the appropriate HRP-conjugated secondary antibody at room temperature for 1 h and then developed using enhanced chemiluminescence (Millipore, Billerica, MA, USA) according to the manufacturer's protocol. The signals were quantified using ImageJ software.

### Plasmid constructs and transfection

To silence endogenous AJUBA, 2 short hairpin RNA (shRNA) oligonucleotides (5′- CCGGACCTGTATCAAG TGCAACAAACTCGAGTTTGTTGCACTTGATACAG GTTTTTTG-3′ and 5′-CCGGGACTTCTCCAACCAAGT ATACCTCGAGGTATACTTGGTTGGAGAAGTCTTTT TG-3′) were cloned into the pLKO.1 vector. For overexpression, the coding DNA sequence (CDS) of AJUBA was cloned into the pLenti-C-Myc-DDK-IRES-Puro expression vector (Origene, MD, USA). The recombined plasmid and the packaging plasmid mixture (Invitrogen, Carlsbad, CA, USA) were co-transfected into HEK 293T cells using Lipofectamine 2000 (Invitrogen). The supernatant containing lentivirus was collected and used to infect ESCC cell lines. The corresponding empty vectors were used as controls. Stable cell lines were selected for 14 d with 0.5 μg/ml puromycin (Invitrogen). The efficiencies of knockdown and overexpression were controlled by detecting the mRNA and protein levels of AJUBA via RT-PCR and Western blot.

### Wound healing assay

The stably transfected KYSE450 and KYSE510 cells were plated in 6-well plates, and the cell monolayer was scratched 24 h later using a 10-μl pipette tip. Wound closure was then monitored 0 h and 24 h after scratching by photographing 9 random fields of the sample, and the change in the area of the wound was calculated using ImageJ software. The experiments were performed in triplicate.

### Migration and invasion assays

Cultured ESCC cells were starved for 24 h before the migration assay. The cells were counted and suspended in 200 μl of serum-free medium and seeded into the upper chamber of a Transwell insert (Corning, New York, NY, USA); 750 μl of 20% FBS 1640 medium was added to the lower chamber. After the cells were cultured for 36 h, cells that had migrated to the bottom surface were fixed with methanol for 2 min and stained with Giemsa for 20 min. An invasion assay was performed with BD BioCoat Matrigel Invasion Chambers following the manufacturer's instructions. Nine random fields from each filter were then imaged, and the cells were counted using ImageJ software. Three independent experiments were conducted for both the migration and invasion assays. For the *in vivo* metastasis assay, two groups of 4- to 5-week-old SCID-Beige mice (9 mice per group) were administered tail-vein injections of 1 × 10^6^ Vec-KYSE450 or AJUBA-KYSE450 cells. Twelve weeks later, the mice were sacrificed, and the tumor nodules onthe lungs were counted, excised and embedded in paraffin for hematoxylin and eosin staining (H&E). The mice used in this study received humane care, and the experiments were performed according to the guidelines approved by the Cancer Institute and Hospital of Chinese Academy of Medical Sciences Institutional Animal Care and Use Committee.

### Cell proliferation assay and xenograft model

To test the growth rate of stably transfected cells, cell counting kit-8 (CCK8) assays were performed according to the manufacturer's instructions. Briefly, 2 × 10^3^ cells were plated into 96-well plates, and cell viability was measured by CCK8 assay (Dojindo, Kumamoto, Japan) and quantified with the SoftMax^®^ Pro 5 program at 0, 24, 48, 72, 96 and 120 h after seeding. For the clone formation assay, 500 cells were seeded in 6-well plates and cultured for 10–14 d in RPMI 1640 medium supplemented with 10% FBS; the surviving colonies were fixed and stained with 1% crystal violet (Amresco, Solon, OH, USA). For the *in vivo* tumorigenicity assay, 5 × 10^6^ sh-AJUBA cells and control cells were injected (s.c.) into the left and right dorsal flanks of female BALB/c-nu mice (4–5 weeks, 15–17 g, Hua fu kang, Beijing, China). Tumor formation was monitored every three days by measuring the tumor size with a caliper. The tumor volume was then calculated using the following formula: V = (L × W^2^)/2. Five weeks later, the animals were sacrificed by cervical dislocation, and the tumors were excised and weighed.

### Induced apoptosis

The ESCC cells were seeded in 6-well plates, cultured in RPMI 1640 medium supplemented with 10% FBS for 24 h and then treated with cisplatin for 24 h. The apoptotic cells were double-labeled with annexin V–fluorescein isothiocyanate (FITC) and propidium iodide (PI) using a BD Pharmingen^™^ PE Annexin V Apoptosis Detection Kit I (Becton Dickinson FACSCanto II, NJ, USA) according to the manufacturer's instructions.

### TMAs and immunohistochemistry

TMAs containing 81 primary esophageal tumors and 60 corresponding non-tumor tissues were retrieved from the archive of paraffin-embedded tissues obtained between 2000 and 2008 at the Cancer Institute and Hospital of the Chinese Academy of Medical Sciences. Briefly, the TMA sections were deparaffinized, rehydrated, and dipped in 3% hydrogen peroxide solution for 30 min. The TMA sections were then treated with 10% goat serum at room temperature for 30 min, followed by incubation with anti-AJUBA (1:300 dilution) at 4°C overnight. After washing with PBS, the TMA sections were incubated with polyperoxidase-anti-rabbit IgG at room temperature for 20 min. DAB and hematoxylin were used to stain and counterstain the sections, respectively. Two independent pathologists analyzed the IHC stains. The staining intensity was graded as follows: 0 (negative), 1 (weak), 2 (moderate) and 3 (strong).

### RNA sequencing

In this study, we used KYSE180, KYSE450 and KYSE510 cells for RNA sequencing detection. For each cell line, 3 wells of 6-well plates were treated with shRNA, and 3 were treated with empty vector. Cells were then harvested 96 h after infection, and knockdown was confirmed by qPCR and Western blot. Total RNA quality analysis, cDNA library preparation and sequencing were carried out by Huada Genomics Co., Ltd. (Shenzhen, China).

### Statistical analysis

Statistical analyses were performed using SPSS 13.0 software. The statistical comparisons between groups were analyzed using Student's *t*-test; the results are expressed as the mean ± SEM. Paired Students’ *t*-test and χ^2^ test were used to analyze the mRNA and protein levels of AJUBA. Correlations between AJUBA mRNA levels and MMP10 or MMP13 mRNA levels were analyzed by Pearson's correlation coefficient. Differences were considered significant when *P* < 0.05.

## SUPPLEMENTARY MATERIALS FIGURES AND TABLES




