# Supplementary material for: AJUBA promotes the migration and invasion of esophageal squamous cell carcinoma cells through upregulation of MMP10 and MMP13 expression
Source: Oncotarget. 2016 May 9;7(24):36407–18. doi: 10.18632/oncotarget.9239 (PMC5095009; doi:10.18632/oncotarget.9239)
Supplement: Supplementary file 1 [file oncotarget-07-36407-s001.pdf]

AJUBA promotes the migration and invasion of esophageal squamous cell carcinoma cells through upregulation of MMP10 and MMP13 expression

Supplementary Materials

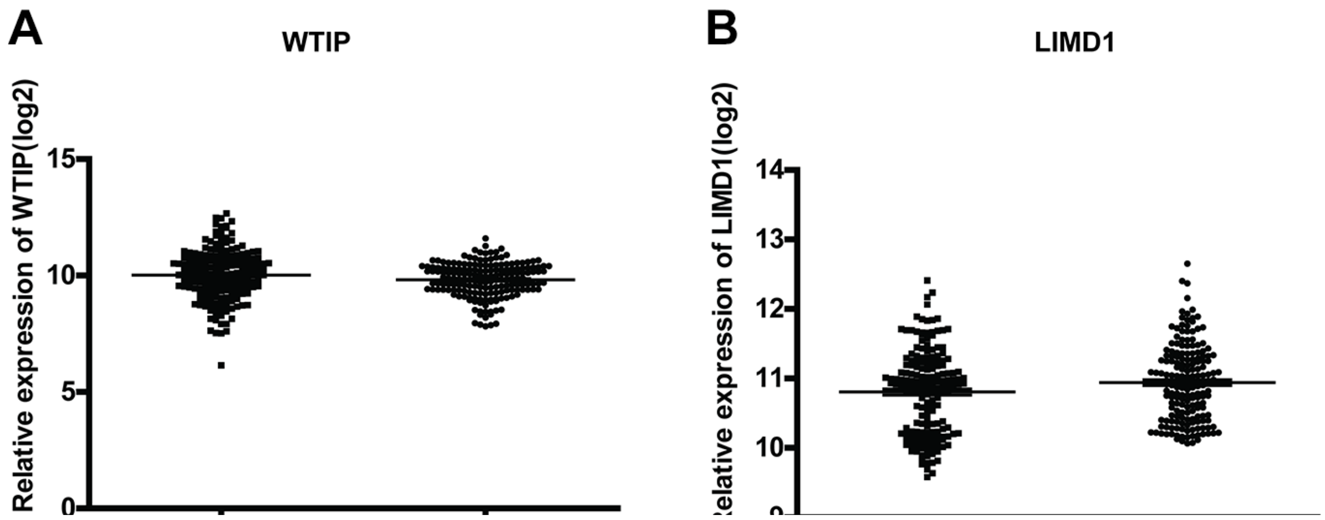

Supplementary Figure S1: The expression levels of WTIP and LIMD1 in 179 pairs of primary ESCC tissues.

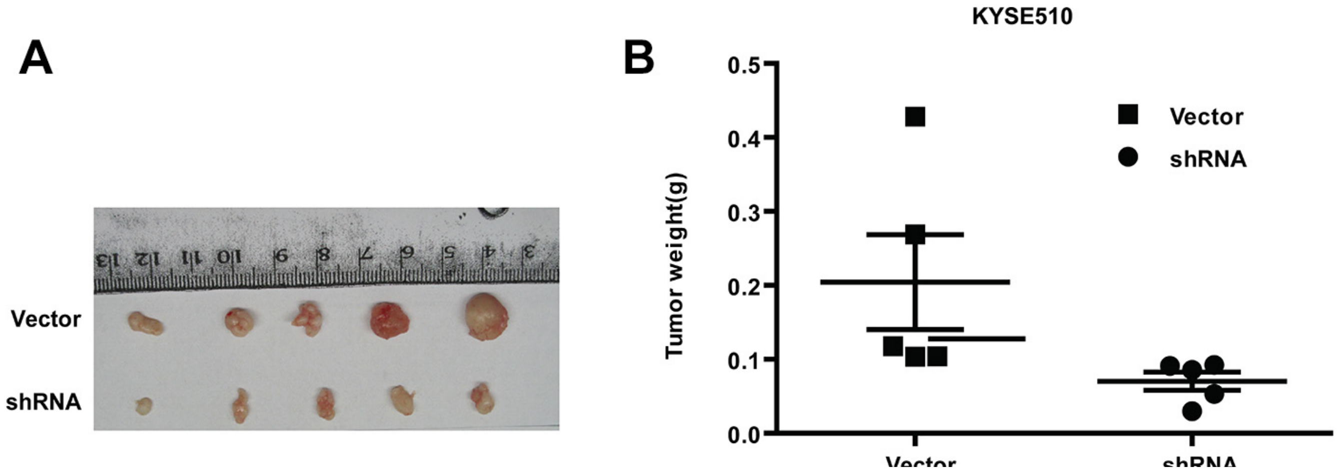

Supplementary Figure S2: Depletion of AJUBA suppressed the tumor growth of KYSE510 cells *in vivo.*

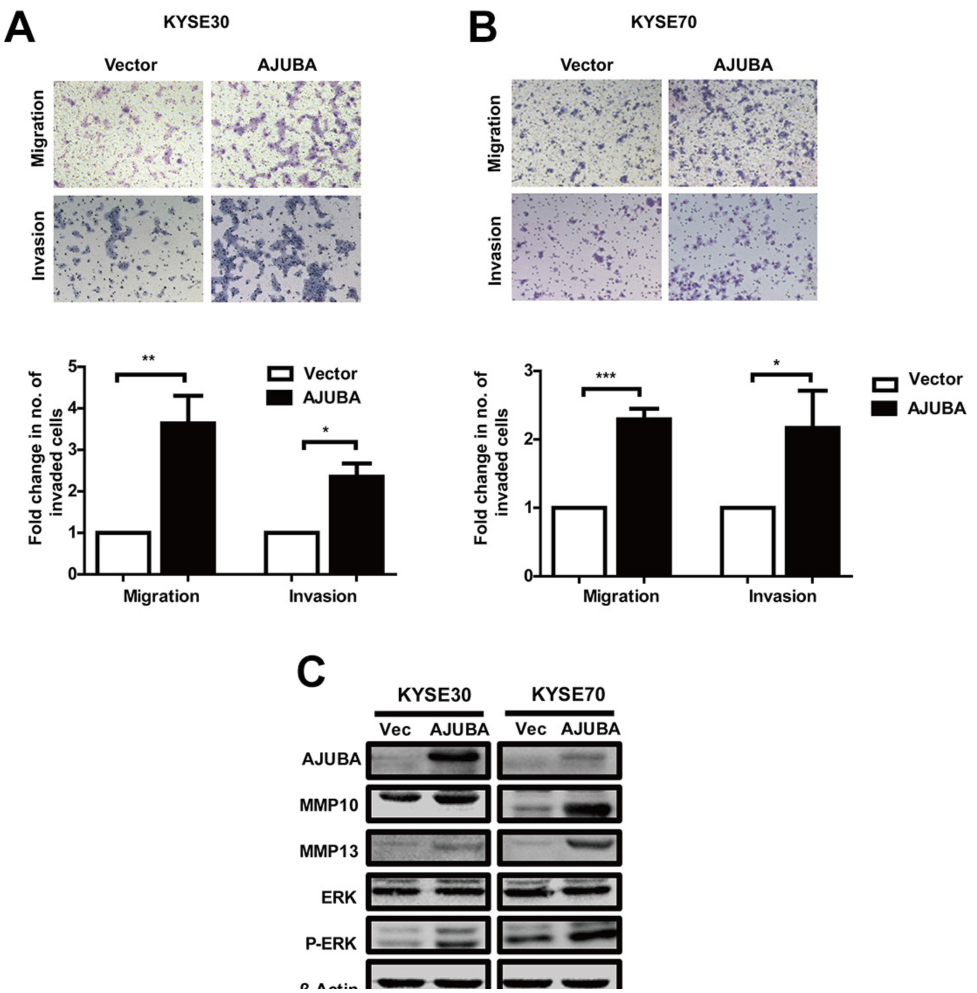

Supplementary Figure S3: AJUBA overexpression increased the motility of KYSE30 and KYSE70 cells.

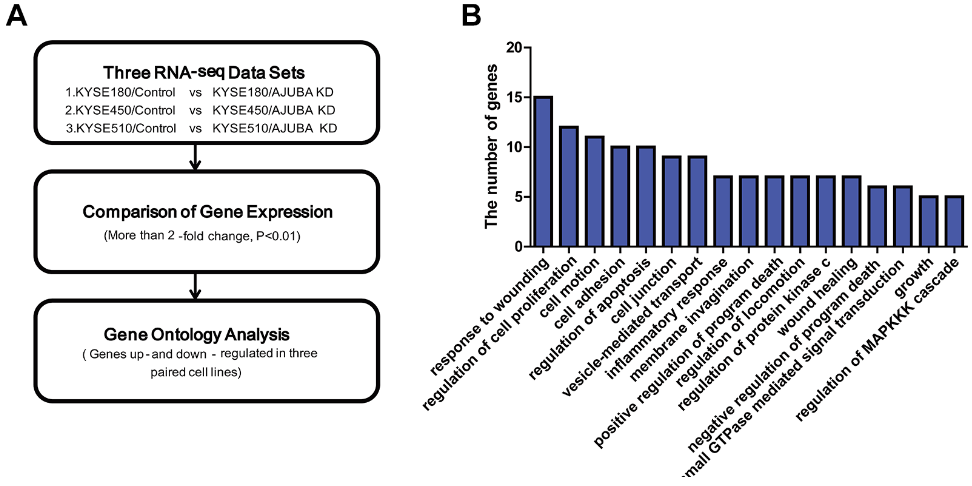

Supplementary Figure S4: The strategy for RNA-sequencing.

Supplementary Table S1: Genes regulated by AJUBA activity in three cell lines. See Supplementary_Table_S1

Supplementary Table S2: Correlation between AJUBA and MMP10 and MMP13 expression in ESCCs

AJUBA expression	Cases	MMP10 expression		P value
		low level (%)	high level (%)	
low level	89	56 (62.9%)	33 (37.1%)	**< 0.001**
high level	90	33(36.7%)	57 (63.3%)	

AJUBA expression	Cases	MMP13 expression		P value
		low level (%)	high level (%)	
low level	89	59 (66.3%)	30 (33.7%)	**< 0.001**
high level	90	30 (33.3%)	60 (66.7%)	

		MMP10	MMP13
AJUBA	Pearson Correlation	.441	.404
	Sig. (2-tailed)	**.000**	**.000**
	Case	179	179

statistical significance ($p < 0.05$) is shown in bold.

Supplementary Table S3: Primers used in this study

GAPDH	
Forward	GAAGGTGAAGGTCGGAGTC
Reverse	GAAGATGGTGATGGGATTTC
AJUBA	
Forward	AGGCCAGGGAGGACTACTTCG
Reverse	GCCTCCTGAAACCCTGAAA
MMP10	
Forward	GGACCTGGGCTTTATGGAGATAT
Reverse	CCCAGGGAGTGGCCAAGT
MMP13	
Forward	TCCCAGGAATTGGTGATAAAGTAGA
Reverse	CTGGCATGACGCGAACAATA
