# Supplementary material for: AJUBA promotes the migration and invasion of esophageal squamous cell carcinoma cells through upregulation of MMP10 and MMP13 expression
Source: Oncotarget. 2016 May 9;7(24):36407–18. doi: 10.18632/oncotarget.9239 (PMC5095009; doi:10.18632/oncotarget.9239)
Supplement: Supplementary file 2 [file oncotarget-07-36407-s002.docx]

Supplementary Table S1: Genes regulated by AJUBA activity in three cell lines

| **EntrezGeneID** | **GeneSymbol** | **180** | **180/shAjuba** | **450** | **450/shAjuba** | **510** | **510/shAjuba** | **logFC** | **Fold change** |  |
| --- | --- | --- | --- | --- | --- | --- | --- | --- | --- | --- |
| 810 | CALML3 | 2.041 | −1.537 | 3.21 | −2.406 | −3.147 | −4.781 | −4.122 | 17.42 | down |
| 3848 | KRT1 | 6.557 | 3.321 | 3.561 | −1.558 | −2.41 | −1.974 | −3.316 | 9.96 | down |
| 4094 | MAF | 1.722 | −0.138 | 4.604 | 1.155 | −0.826 | −3.196 | −2.893 | 7.43 | down |
| 196913 | C14orf183 | −0.348 | −3.411 | 0.412 | −1.92 | −1.562 | −4.781 | −2.779 | 6.86 | down |
| 401134 | LOC401134 | −2.185 | −4.996 | 0.788 | −2.406 | −1.562 | −3.196 | −2.643 | 6.25 | down |
| 374454 | KRT77 | 2.019 | −0.604 | −0.099 | −3.143 | −1.562 | −3.196 | −2.531 | 5.78 | down |
| 4319 | MMP10 | 5.853 | 3.348 | 6.299 | 3.728 | −2.41 | −3.196 | −2.486 | 5.60 | down |
| 1960 | EGR3 | 5.211 | 2.868 | 2.605 | −0.335 | 3.251 | 0.773 | −2.474 | 5.56 | down |
| 4322 | MMP13 | 1.878 | 0.558 | 7.453 | 4.606 | −1.562 | −1.322 | −2.471 | 5.54 | down |
| 768 | CA9 | −0.134 | −1.537 | −1.055 | −4.728 | 4.342 | 1.789 | −2.462 | 5.51 | down |
| 2568 | GABRP | −1.085 | −3.411 | −1.973 | −4.728 | 0.822 | −1.322 | −2.335 | 5.05 | down |
| 80035 | ANP32A-IT1 | −0.237 | −2.189 | 0.923 | −1.027 | 0.882 | −1.974 | −2.244 | 4.74 | down |
| 3866 | KRT15 | 8.216 | 7.182 | 10.207 | 6.804 | 9.359 | 7.366 | −2.138 | 4.40 | down |
| 1959 | EGR2 | 5.479 | 3.955 | 5.082 | 2.756 | 4.888 | 2.476 | −2.002 | 4.00 | down |
| 7358 | UGDH | 6.559 | 4.692 | 7.842 | 4.922 | 7.769 | 6.868 | −1.873 | 3.66 | down |
| 1961 | EGR4 | 2.148 | 0.361 | −0.285 | −1.92 | 0.553 | −1.611 | −1.847 | 3.60 | down |
| 1556 | CYP2B7P | −0.348 | −2.189 | −2.821 | −2.406 | 5.635 | 3.69 | −1.798 | 3.48 | down |
| 2591 | GALNT3 | 5.521 | 4.221 | 5.71 | 3.661 | 7.292 | 5.47 | −1.721 | 3.30 | down |
| 79755 | ZNF750 | 3.78 | 2.123 | 0.529 | −0.204 | 2.616 | 0.263 | −1.688 | 3.22 | down |
| 767558 | LUZP6 | 7.463 | 6.134 | 7.324 | 5.461 | 7.881 | 6.114 | −1.65 | 3.14 | down |
| 7045 | TGFBI | −3.407 | −3.411 | 0.215 | −2.406 | 9.677 | 8.037 | −1.643 | 3.12 | down |
| 2196 | FAT2 | 8.401 | 7.229 | 9.404 | 7.446 | 8.834 | 7.121 | −1.613 | 3.06 | down |
| 11057 | ABHD2 | 6.6 | 5.087 | 6.541 | 4.9 | 6.796 | 5.278 | −1.556 | 2.94 | down |
| 4922 | NTS | 6.221 | 4.723 | 2.851 | 0.887 | −1.562 | −1.974 | −1.531 | 2.89 | down |
| 2354 | FOSB | 8.688 | 6.762 | 7.061 | 5.696 | 8.377 | 7.311 | −1.455 | 2.74 | down |
| 100129550 | LOC100129550 | 5.231 | 3.68 | 3.353 | 1.748 | 3.472 | 2.358 | −1.453 | 2.74 | down |
| 257177 | C1orf192 | 3.698 | 2.88 | 3.814 | 2.306 | 4.347 | 2.338 | −1.426 | 2.69 | down |
| 163351 | GBP6 | 7.037 | 5.97 | 6.64 | 4.762 | −0.209 | −0.533 | −1.404 | 2.65 | down |
| 92270 | ATP6AP1L | 1.974 | 0.619 | 2.45 | 1.381 | 3.132 | 1.448 | −1.384 | 2.61 | down |
| 399948 | COLCA1 | 1.608 | 0.676 | 2.095 | 0.63 | 4.309 | 2.796 | −1.378 | 2.60 | down |
| 1827 | RCAN1 | 5.423 | 4.001 | 4.339 | 2.819 | 2.448 | 1.559 | −1.372 | 2.59 | down |
| 3164 | NR4A1 | 8.279 | 6.902 | 7.08 | 5.855 | 7.427 | 5.93 | −1.367 | 2.58 | down |
| 246312 | C21orf91-OT1 | 2.991 | 1.772 | 4.759 | 3.209 | 4.704 | 3.471 | −1.355 | 2.56 | down |
| 9902 | MRC2 | 6.491 | 5.15 | 5.222 | 3.898 | 2.975 | 1.594 | −1.34 | 2.53 | down |
| 92597 | MOB1B | 5.471 | 4.398 | 5.599 | 4.127 | 4.994 | 3.462 | −1.338 | 2.53 | down |
| 4854 | NOTCH3 | 8.272 | 7.098 | 4.959 | 3.427 | 7.498 | 6.182 | −1.307 | 2.47 | down |
| 19 | ABCA1 | 5.167 | 3.722 | 1.625 | −0.204 | 2.487 | 1.877 | −1.307 | 2.47 | down |
| 79800 | CARF | 3.251 | 2.143 | 3.837 | 2.093 | 3.492 | 2.457 | −1.303 | 2.47 | down |
| 30819 | KCNIP2 | 1.903 | 0.496 | 2.721 | 1.203 | 2.186 | 1.241 | −1.298 | 2.46 | down |
| 324 | APC | 5.544 | 4.518 | 5.67 | 4.363 | 5.483 | 3.895 | −1.292 | 2.45 | down |
| 100506211 | MIR210HG | 4.478 | 3.228 | 3.032 | 1.959 | 1.496 | −0.533 | −1.292 | 2.45 | down |
| 84985 | FAM83A | −1.533 | −1.826 | −2.336 | −3.143 | 7.33 | 5.959 | −1.29 | 2.45 | down |
| 10804 | GJB6 | 7.023 | 5.597 | 3.453 | 2.167 | 2.616 | 2.013 | −1.267 | 2.41 | down |
| 1829 | DSG2 | 8.847 | 8.053 | 9.055 | 7.378 | 8.628 | 7.305 | −1.265 | 2.40 | down |
| 646 | BNC1 | 3.251 | 1.923 | 3.052 | 1.462 | 4.601 | 3.545 | −1.255 | 2.39 | down |
| 11037 | STON1 | 6.139 | 5.215 | 5.787 | 3.976 | −0.089 | −0.026 | −1.25 | 2.38 | down |
| 284018 | C17orf58 | 4.19 | 2.52 | 4.126 | 2.994 | 3.307 | 2.495 | −1.242 | 2.37 | down |
| 5054 | SERPINE1 | 3.875 | 2.204 | 0.349 | −0.084 | 4.42 | 3.353 | −1.234 | 2.35 | down |
| 9404 | LPXN | 1.927 | 0.785 | 3.304 | 1.812 | 3.317 | 2.296 | −1.225 | 2.34 | down |
| 143888 | KDELC2 | 6.315 | 5.008 | 5.424 | 4.435 | 4.61 | 3.224 | −1.216 | 2.32 | down |
| 51208 | CLDN18 | 1.484 | 0.048 | 3.743 | 2.673 | −0.089 | −1.611 | −1.209 | 2.31 | down |
| 84962 | AJUBA | 8.138 | 7.135 | 7.251 | 5.536 | 8.331 | 7.449 | −1.192 | 2.28 | down |
| 54149 | C21orf91 | 3.589 | 2.711 | 5.372 | 3.962 | 5.249 | 4.098 | −1.19 | 2.28 | down |
| 1176 | AP3S1 | 5.425 | 4.319 | 5.338 | 4.121 | 6.413 | 5.212 | −1.175 | 2.26 | down |
| 57198 | ATP8B2 | 6.177 | 5.255 | 5.211 | 3.695 | 3.539 | 2.438 | −1.154 | 2.22 | down |
| 2938 | GSTA1 | 5.938 | 4.81 | −1.684 | −3.143 | −1.925 | −3.196 | −1.152 | 2.22 | down |
| 59338 | PLEKHA1 | 7.375 | 6.243 | 6.625 | 5.193 | 6.325 | 5.446 | −1.148 | 2.22 | down |
| 4015 | LOX | 2.284 | 1.379 | 3.952 | 2.167 | 5.073 | 4.205 | −1.14 | 2.20 | down |
| 100652770 | LOC100652770 | 8.294 | 7.5 | 8.41 | 6.907 | 8.06 | 6.954 | −1.134 | 2.19 | down |
| 27245 | AHDC1 | 5.485 | 4.372 | 5.334 | 3.854 | 5.37 | 4.534 | −1.132 | 2.19 | down |
| 4548 | MTR | 6.535 | 5.86 | 6.203 | 5.026 | 6.434 | 4.907 | −1.111 | 2.16 | down |
| 5784 | PTPN14 | 7.067 | 6.486 | 6.583 | 5.059 | 7.802 | 6.54 | −1.109 | 2.16 | down |
| 9866 | TRIM66 | 4.895 | 4.067 | 5.841 | 4.667 | 4.537 | 3.167 | −1.103 | 2.15 | down |
| 54626 | HES2 | 4.286 | 3.266 | 2.114 | 0.227 | 3.849 | 2.954 | −1.1 | 2.14 | down |
| 1371 | CPOX | 5.797 | 4.583 | 5.187 | 3.891 | 5.512 | 4.705 | −1.099 | 2.14 | down |
| 829 | CAPZA1 | 7.763 | 6.931 | 7.544 | 6.32 | 7.561 | 6.319 | −1.098 | 2.14 | down |
| 401474 | SAMD12 | 5.651 | 4.602 | 4.069 | 3.112 | 8.524 | 7.33 | −1.095 | 2.14 | down |
| 169611 | OLFML2A | 6.133 | 4.74 | 4.927 | 3.695 | 4.213 | 3.785 | −1.094 | 2.13 | down |
| 2353 | FOS | 9.049 | 8.055 | 7.369 | 5.981 | 9.287 | 8.423 | −1.081 | 2.12 | down |
| 5874 | RAB27B | 2.673 | 1.413 | 2.188 | 1.054 | 3.985 | 3.033 | −1.08 | 2.11 | down |
| 10905 | MAN1A2 | 6.068 | 5.433 | 5.544 | 4.182 | 4.959 | 3.563 | −1.07 | 2.10 | down |
| 642587 | MIR205HG | 8.315 | 6.931 | 8.312 | 7.092 | 8.317 | 7.73 | −1.064 | 2.09 | down |
| 100302692 | FTX | 6.677 | 6.053 | 6.133 | 4.663 | 6.759 | 5.633 | −1.052 | 2.07 | down |
| 78990 | OTUB2 | 2.729 | 1.574 | 3.201 | 2.284 | 2.009 | 0.891 | −1.045 | 2.06 | down |
| 5163 | PDK1 | 5.6 | 4.549 | 4.656 | 3.197 | 5.105 | 4.381 | −1.045 | 2.06 | down |
| 257106 | ARHGAP30 | 3.352 | 2.299 | 1.427 | 0.63 | 3.251 | 2.114 | −1.036 | 2.05 | down |
| 836 | CASP3 | 5.544 | 4.628 | 4.685 | 3.59 | 6.317 | 5.245 | −1.022 | 2.03 | down |
| 261729 | STEAP2 | 4.268 | 3.687 | 6.821 | 5.493 | 3.392 | 2.495 | −1.022 | 2.03 | down |
| 200895 | DHFRL1 | 3.142 | 2.28 | 3.687 | 2.349 | 3.558 | 2.719 | −1.017 | 2.02 | down |
| 56894 | AGPAT3 | 6.103 | 5.036 | 6.388 | 5.557 | 6.807 | 5.669 | −1.013 | 2.02 | down |
| 7078 | TIMP3 | 0.563 | −0.241 | 1.955 | 1.25 | 6.05 | 4.952 | −1.006 | 2.01 | down |
| 27250 | PDCD4 | 7.199 | 6.065 | 6.719 | 5.416 | 6.437 | 5.862 | −1.006 | 2.01 | down |
| 7334 | UBE2N | 6.791 | 5.822 | 6.817 | 5.832 | 6.759 | 5.698 | −1.005 | 2.01 | down |
| 3383 | ICAM1 | 1.75 | 2.504 | −0.014 | 1.25 | 4.828 | 5.862 | 1 | 2.00 | up |
| 80008 | TMEM156 | 2.063 | 2.905 | 4.552 | 5.646 | 3.841 | 4.821 | 1.011 | 2.02 | up |
| 161582 | DYX1C1 | 1.608 | 2.779 | 3.129 | 4.224 | 2.448 | 3.213 | 1.018 | 2.03 | up |
| 1846 | DUSP4 | 0.841 | 2.353 | −1.973 | −0.48 | 5.121 | 5.996 | 1.024 | 2.03 | up |
| 79847 | TMEM180 | 3.122 | 3.762 | 3.321 | 4.551 | 2.427 | 3.666 | 1.035 | 2.05 | up |
| 8507 | ENC1 | 3.656 | 4.806 | 3.11 | 4.42 | 3.094 | 3.674 | 1.058 | 2.08 | up |
| 79605 | PGBD5 | 0.939 | 1.69 | 0.472 | 1.959 | 3.392 | 4.426 | 1.065 | 2.09 | up |
| 84984 | CEP19 | 2.492 | 3.687 | 2.605 | 3.278 | 2.634 | 3.95 | 1.069 | 2.10 | up |
| 3162 | HMOX1 | 2.835 | 4.156 | 0.879 | 2.328 | 4.195 | 4.995 | 1.081 | 2.12 | up |
| 6286 | S100P | −2.185 | 0.048 | −2.336 | −1.92 | 7.466 | 8.528 | 1.104 | 2.15 | up |
| 306 | ANXA3 | 1.547 | 3.33 | 4.816 | 5.664 | 6.633 | 7.782 | 1.114 | 2.16 | up |
| 27293 | SMPDL3B | 0.68 | 1.633 | 2.734 | 4.083 | 2.467 | 3.423 | 1.143 | 2.21 | up |
| 729967 | MORN2 | 3.597 | 4.522 | 3.749 | 5.228 | 3.576 | 4.579 | 1.159 | 2.23 | up |
| 9076 | CLDN1 | 0.563 | 1.48 | 5.518 | 6.359 | 5.464 | 6.985 | 1.162 | 2.24 | up |
| 89846 | FGD3 | 2.956 | 4.023 | 0.529 | 1.339 | 5.517 | 6.811 | 1.181 | 2.27 | up |
| 92558 | CCDC64 | 3.799 | 4.933 | 3.156 | 4.475 | 4.984 | 6.168 | 1.203 | 2.30 | up |
| 53842 | CLDN22 | 3.604 | 4.631 | 1.934 | 3.386 | 2.279 | 3.615 | 1.217 | 2.32 | up |
| 124935 | SLC43A2 | 3.036 | 4.529 | 4.433 | 5.956 | 3.779 | 4.406 | 1.263 | 2.40 | up |
| 57158 | JPH2 | −2.185 | −1.537 | −3.558 | −2.406 | 3.144 | 4.511 | 1.282 | 2.43 | up |
| 7424 | VEGFC | 1.903 | 2.843 | 0.349 | 1.78 | 2.036 | 3.607 | 1.304 | 2.47 | up |
| 404217 | CTXN1 | 3.232 | 4.31 | 3.081 | 4.713 | 3.251 | 4.445 | 1.311 | 2.48 | up |
| 478 | ATP1A3 | 3.334 | 4.826 | 3.032 | 4.352 | −0.826 | −0.874 | 1.326 | 2.51 | up |
| 148252 | DIRAS1 | 3.352 | 4.96 | 2.223 | 3.255 | −2.41 | −2.459 | 1.34 | 2.53 | up |
| 397 | ARHGDIB | 0.137 | 1.512 | −0.619 | 1.422 | 5.545 | 6.813 | 1.356 | 2.56 | up |
| 1543 | CYP1A1 | 5.46 | 6.619 | 2.658 | 5.138 | 4.276 | 5.2 | 1.417 | 2.67 | up |
| 2921 | CXCL3 | 1.383 | 2.725 | 2.551 | 3.983 | 0.397 | 1.96 | 1.43 | 2.69 | up |
| 283692 | LOC283692 | 1.878 | 4.089 | 4.58 | 5.651 | −0.089 | 1.448 | 1.43 | 2.69 | up |
| 51083 | GAL | 2.148 | 3.339 | 4.762 | 6.342 | −4.732 | −4.781 | 1.439 | 2.71 | up |
| 9479 | MAPK8IP1 | 2.822 | 4.278 | 3.609 | 5.032 | 1.049 | 2.513 | 1.441 | 2.72 | up |
| 5010 | CLDN11 | 3.67 | 5.485 | 3.147 | 4.44 | 0.759 | 0.711 | 1.444 | 2.72 | up |
| 8120 | AP3B2 | 2.063 | 4.278 | 4.747 | 5.82 | 0.222 | 1.848 | 1.452 | 2.74 | up |
| 387104 | SOGA3 | −1.822 | 0.289 | 3.814 | 5.029 | 0.222 | 2.358 | 1.453 | 2.74 | up |
| 6678 | SPARC | −1.085 | −1.296 | −3.558 | −4.728 | 7.224 | 8.995 | 1.566 | 2.96 | up |
| 94032 | CAMK2N2 | 1.722 | 3.409 | 1.976 | 3.823 | 0.694 | 1.789 | 1.645 | 3.13 | up |
| 90668 | LRRC16B | 1.695 | 3.492 | 0.412 | 1.715 | −1.925 | −0.137 | 1.651 | 3.14 | up |
| 146206 | RLTPR | 0.985 | 2.711 | 1.8 | 3.927 | 0.553 | 1.448 | 1.761 | 3.39 | up |
| 284339 | TMEM145 | 2.019 | 3.728 | −1.055 | 1.462 | −1.925 | −1.081 | 1.782 | 3.44 | up |
| 9729 | KIAA0408 | −2.185 | −0.241 | 2.851 | 4.314 | −1.562 | 1.758 | 1.802 | 3.49 | up |
| 56892 | C8orf4 | 3.677 | 4.774 | 1.232 | 3.407 | 3.472 | 6.03 | 1.918 | 3.78 | up |
| 90249 | UNC5A | 1.695 | 3.608 | 0.215 | 1.576 | 0.822 | 3.301 | 1.974 | 3.93 | up |
| 256472 | TMEM151A | 0.052 | 1.923 | −0.751 | 1.501 | −4.732 | −3.196 | 1.978 | 3.94 | up |
| 130612 | TMEM198 | 1.03 | 3.189 | 2.076 | 4.399 | 1.334 | 2.603 | 2.023 | 4.06 | up |
| 3785 | KCNQ2 | 0.563 | 2.242 | −0.189 | 2.306 | −4.732 | −2.459 | 2.081 | 4.23 | up |
| 50861 | STMN3 | 4.572 | 6.249 | 3.228 | 6.235 | 1.607 | 3.589 | 2.243 | 4.73 | up |
| 85455 | DISP2 | 0.891 | 2.893 | 0.349 | 2.819 | −1.925 | 0.504 | 2.254 | 4.77 | up |
| 6515 | SLC2A3 | 0.939 | 3.547 | −1.684 | 0.764 | −2.41 | −2.459 | 2.255 | 4.77 | up |
| 401647 | GOLGA7B | 1.695 | 3.637 | −1.055 | 2.349 | 0.022 | 2.338 | 2.367 | 5.16 | up |
| 55118 | CRTAC1 | 1.237 | 3.275 | −1.055 | 2.191 | −0.484 | 2.185 | 2.5 | 5.66 | up |
| 7425 | VGF | −0.134 | 0.981 | −0.285 | 2.966 | −0.34 | 2.495 | 2.528 | 5.77 | up |
| 100131138 | LINC01405 | −3.407 | −0.748 | −5.143 | −2.406 | −3.147 | −0.874 | 2.54 | 5.82 | up |
| 338557 | FFAR4 | −3.407 | −0.748 | −5.143 | −2.406 | −4.732 | −2.459 | 2.568 | 5.93 | up |
| 6543 | SLC8A2 | −0.904 | 2.081 | −2.821 | −1.268 | −4.732 | −1.974 | 2.586 | 6.00 | up |
| 2348 | FOLR1 | −1.292 | 2.37 | −1.443 | 0.027 | −1.273 | 1.15 | 2.676 | 6.39 | up |
| 6855 | SYP | 1.158 | 4.04 | 0.879 | 4.182 | 0.022 | 1.369 | 2.794 | 6.93 | up |
| 23542 | MAPK8IP2 | 0.5 | 3.84 | 0.74 | 4.077 | −1.273 | −2.459 | 2.919 | 7.57 | up |
| 7857 | SCG2 | 3.582 | 7.443 | 3.11 | 4.762 | −1.925 | −1.081 | 2.928 | 7.61 | up |
| 205860 | TRIML2 | −3.407 | −0.909 | −5.143 | −1.027 | 0.477 | 3.258 | 2.935 | 7.65 | up |
| 10537 | UBD | 0.293 | 1.849 | −0.189 | 4.051 | −0.826 | 2.185 | 3.112 | 8.64 | up |
| 1114 | CHGB | 1.03 | 4.407 | −1.055 | 2.656 | −1.925 | −0.533 | 3.205 | 9.22 | up |
| 3569 | IL6 | −2.67 | 0.213 | −2.821 | −0.64 | −0.484 | 3.343 | 3.243 | 9.47 | up |
| 100506178 | LOC100506178 | −4.992 | −3.411 | −5.143 | −0.335 | −3.147 | 0.834 | 3.607 | 12.18 | up |
| 250 | ALPP | −4.992 | 0.43 | −3.558 | 2.69 | 0.126 | 3.859 | 4.775 | 27.37 | up |
